# Prevalence and Progression of Vitamin D Deficiency in Greater Beirut and Mount Lebanon From 2013 to 2022: An Analysis of 19,452 Adults

**DOI:** 10.1002/jcla.70023

**Published:** 2025-03-28

**Authors:** Pia Chedid, Elie Salem‐Sokhn, Said El Shamieh, Rajaa Fakhoury

**Affiliations:** ^1^ Molecular Testing Laboratory, Department of Medical Laboratory Technology Faculty of Health Sciences, Beirut Arab University Beirut Lebanon

**Keywords:** Greater Beirut, Mount Lebanon, prevalence, urban areas, vitamin D deficiency, vitamin D levels

## Abstract

**Aim:**

This study aimed to describe the prevalence and progression of vitamin D (VitD) deficiency in a large sample of Lebanese adults.

**Methods:**

A retrospective analysis of 19,452 medical records of Lebanese adults primarily residing in Greater Beirut and Mount Lebanon was included. Serum VitD levels were measured using a chemiluminescent assay.

**Results:**

Overall, 31% of our participants were deficient (< 20 ng/mL), 28% were insufficient (20–30 ng/mL), and 41% were sufficient (> 30 ng/mL) for VitD. The overall average VitD levels were 26 ± 12 ng/mL (min: 3 ng/mL, max: 220 ng/mL). While 40% of participants below 35 years old presented with VitD level deficiency, this level decreased significantly by 13% and 11% in age groups 55–65 and above 65 years old, respectively (*p* < 0.01). Females had a 35% lower risk of VitD deficiency than males (OR = 0.65, *p* < 0.01). Second, living in the greater Beirut region increased the risk of developing VitD deficiency by 41% (OR = 1.408, *p* < 0.01). Finally, living in a region with moderate (OR = 1.33, *p* < 0.11) to high pollution (OR = 1.45, *p* < 0.01) increased the risk by 33% and 45%, respectively. The number of individuals referred for testing VitD level in our tertiary healthcare center increased four times in the past 10 years, from 1120 in 2013 to 4633 in 2022. This observation is correlated with higher VitD levels and, thus, a significant decrease in the trend of VitD deficiency in 2022 compared to the previous years (*p* < 0.001).

**Conclusion:**

The prevalence of VitD deficiency has decreased over the past 10 years in Greater Beirut and Mount Lebanon.

## Graphical abstract

Overall, 31% of our participants were deficient, 28% were insufficient, and 41% were sufficient. While 40% of participants below 35 years old presented with VitD deficiency, this level decreased significantly by 13% in older ages (more than 55 years old, *p* < 0.001). Females had a 35% lower risk of VitD deficiency than males (OR = 0.654, *p* < 0.001). Second, living in the greater Beirut region increased the risk of developing VitD deficiency by 41% (OR = 1.408, *p* < 0.001). The number of individuals referred for testing VitD level in our tertiary healthcare center increased four times in the past ten years, from 1120 in 2013 to 4633 in 2022. This observation is correlated with higher VitD levels and, thus, a significant decrease in the trend of VitD deficiency in 2022 compared to the previous years (24%, *p* < 0.001).

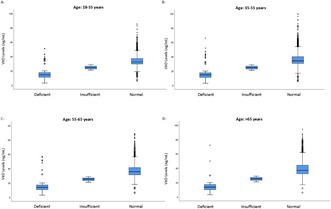

## 1 Introduction

Vitamin D (VitD) is a group of fat‐soluble secosteroids responsible for improving intestinal absorption of calcium, magnesium, iron, zinc, and phosphate [1]. It is essential for preserving bone health, boosting the immune system, and controlling various physiological processes within the body [1]. The metabolism of VitD is a complex process that involves several steps and primarily takes place in the skin, liver, and kidneys [2, 3]. The initial step in VitD metabolism occurs when the skin is exposed to ultraviolet B (UVB) sunlight. UVB radiation converts 7‐dehydrocholesterol, a cholesterol precursor in the skin, into cholecalciferol, also known as vitamin D3 [2, 3]. VitD can also be obtained through dietary sources, including fatty fish, dairy products, and supplements [2, 3]. Once synthesized in the skin or absorbed from the diet, VitD is transported in the bloodstream to a specific carrier protein called vitamin D‐binding protein (DBP) [2, 3]. VitD is first hydroxylated in the liver by 25‐hydroxylase, which converts it into 25‐hydroxyvitamin D, often abbreviated as 25(OH)D or calcidiol. The 25(OH)D is the primary circulating form of VitD and is a marker of a person's VitD status [2, 3]. The second and crucial hydroxylation step occurs in the kidneys, where 25(OH)D is further converted into its active form, 1,25‐dihydroxyvitamin D, often abbreviated as 1,25(OH)_2_D or calcitriol.

The terminology used to describe 25(OH)D levels varies, but it is crucial to define these thresholds explicitly for clarity and consistency. VitD levels are considered deficient with levels < 20 ng/mL, insufficient with levels between 20 and 29 ng/mL, and sufficient with levels > 30 ng/mL [4]. While practice guidelines published in 2024 by the Endocrine Society no longer endorse specific 25(OH)D levels to define VitD sufficiency, insufficiency, and deficiency [5], the clinical measurement of VitD levels, specifically through the assessment of serum 25(OH)D, remains a subject of extensive research, leading to the establishment of various cutoff values that define deficiency, insufficiency, and sufficiency. The consensus on these cutoff values has evolved, with different studies proposing varying thresholds based on health outcomes and population‐specific factors. Recent literature indicates that a 25(OH)D level of less than 20 ng/mL (50 nmol/L) is widely accepted as indicative of VitD deficiency, while levels between 20 and 29 ng/mL (50–74 nmol/L) are often classified as insufficient [6]. For optimal health, many guidelines suggest that serum 25(OH)D levels should exceed 30 ng/mL (75 nmol/L) to ensure adequate calcium absorption and bone health [7]. Notably, the Endocrine Society recommends maintaining levels above 40 ng/mL (100 nmol/L) for optimal health outcomes, particularly in populations at risk for deficiency [7]. In 2023, the World Health Organization in Lebanon [8] adopted and recommended the dissemination of the national guidelines on VitD developed by the American University of Beirut [9].

The current opinion on VitD emphasizes targeted screening rather than universal screening, with specific groups like children, pregnant women, patients on bone‐active drugs, and those with documented hypovitaminosis D benefiting from supplementation without the need for screening [10]. The United States Preventive Services Task Force (USPSTF) indicates a lack of adequate evidence to support the screening of asymptomatic adults, especially those who are not hospitalized or do not have underlying medical conditions [6]. Concentrations of serum VitD that fall below 20 ng/mL are classified as deficient, underscoring the significance of averting and managing such deficiencies [11].

Skeletal health has long been associated with VitD, but during the past 10 years, several studies have shown that VitD also has positive effects on extraskeletal tissues [12]. VitD aids in controlling cell growth and halting the spread of cancer [13], and based on literature, higher levels have been linked to lower cancer incidence and cancer‐related mortality [12]. In addition, one of the most crucial hormones for maintaining blood pressure, renin, was shown to be produced under the control of VitD [14]. As a result, a VitD deficiency may speed up the onset and progression of cardiovascular disease and hypertension [15]. Epidemiological studies have shown that VitD might play a multifaceted role in modulating bacterial growth and survival, which has significant implications for infection prevention and treatment [16, 17]. According to the literature, the risk factors for VitD deficiency include air pollution, season, sunscreen use, latitude, diet, sedentary jobs, and others [18].

In recent years, there has been a sharp rise in the number of new studies looking into the incidence of VitD deficiency in 81 countries [18]. A recent systematic review also confirmed the high prevalence of low VitD levels in Southern Europe and the Eastern Mediterranean regions [19]. Another study noted that the prevalence of VitD deficiency in Asia has risen between 2008 and 2014. While the exact cause of the decline in VitD levels remains uncertain, factors such as increased urbanization, air pollution, and reduced outdoor activity may contribute to this trend [20]. Beirut and Mount Lebanon are pivotal regions within Lebanon. With a latitude of 33.8886288 and a longitude of 35.4954794, Beirut, Lebanon, is elevated to a height of around 100 m (328 ft) [21]. Beirut is well‐known for having an abundance of sunshine; on average, there are 2940 h of sunshine every year, or roughly 8 h and 2 min per day. The city has about 300 sunny days a year, with long, vibrant summer days [22]. Mount Lebanon has peaks that rise as high as 3088 m (10,131 ft), and it is approximately located at latitude 34°17′60.00″ N and longitude 36°06′ 60.00″ E. As Mount Lebanon is at a higher altitude and has a cooler climate than Beirut, it experiences fewer sunny days yearly [23, 24]. Due to its coastal position, Beirut's diet is mostly composed of various seafood and a well‐balanced mix of meats, including lamb, chicken, and cattle. In contrast, because of its inland and steep location, Mount Lebanon's traditional diet consists primarily of meats, particularly lamb and chicken. Seafood is less prevalent in Mount Lebanon compared to Beirut [25]. Since data on the prevalence of VitD deficiency in Lebanon are scarce, this study aimed to determine the prevalence of VitD deficiency in the Greater Beirut and Mount Lebanon regions over 10 years, from 2013 to 2022.

## 2 Materials and Methods

### 2.1 Study Population

This retrospective cohort study was conducted at the Lebanese Hospital Geitaoui‐University Medical Center in Beirut. A retrospective analysis of data collected from medical laboratory records was conducted after obtaining the hospital's institutional review board approval (IRB: 2023‐IRB‐017), and 19,452 participants' records were analyzed between January 2013 and December 2022.

### 2.2 Data Collection

VitD measurement was conducted with blood serum using a Roche Cobas 6000 analyzer. To ensure consistent VitD results over the study period, the instrument typically underwent regular calibration and quality control procedures according to manufacturer guidelines to ensure the accuracy and reliability of measurements. Calibration involves setting reference points to ensure accurate readings, and controls are used to monitor consistency. Roche periodically updates assays to improve performance, sensitivity, and specificity. For the calibrator traceability, the calibrator used in Roche systems is typically traceable to international standards. During the study period, if there were changes in calibrators, Roche would provide information on traceability and any implications for result interpretation. VitD samples were ideally analyzed shortly after collection to minimize degradation. When immediate analysis was not possible, samples were refrigerated to preserve stability.

We categorized VitD levels using two classifications. The first classification defined deficiency, insufficiency, and sufficiency based on thresholds of < 20 ng/mL, 21–29 ng/mL, and > 30 ng/mL, respectively [26]. The second classification utilized the WHO for the Eastern Mediterranean Region. It is a binary approach, categorizing levels as either sufficient (≥ 20 ng/mL) or insufficient/deficient (< 20 ng/mL) [8].

In addition, the age, gender, and region of each patient were collected and thus analyzed along with pollution levels (low, moderate, high) and seasonal changes (summer, autumn, winter, and spring). This study employed air quality data from AccuWeather [27] to evaluate pollution levels across diverse regions in Lebanon. AccuWeather's platform integrates air quality metrics into its weather forecasting services, offering localized, precise insights into environmental pollution levels. Data collection involved retrieving Air Quality Index (AQI) values and specific pollutant concentrations—including PM2.5, PM10, NO2, SO2, and O3—from AccuWeather's database for designated locations representing Beirut, Mount Lebanon, and other Lebanese regions. The pollution color codes red, orange/yellow, and green were coded as high, moderate, and low pollution levels, respectively.

### 2.3 Statistical Analyses

The analyses were conducted using SPSS software version 20 (SPSS Inc., IL, USA). Continuous variables were presented as mean values ± standard deviations, and categorical variables were given as numbers and percentages. The chi‐squared test (*χ* ^2^), Kruskal–Wallis *H*, and Mann–Whitney *U* tests were performed to search for significant differences between categorical and continuous variables (Mann–whitney *U* test to compare two study groups, Kruskal–Wallis *H* to compare three study groups). To test the association with VitD deficiency, a binary multiple logistic regression model was used while correcting for several confounders (age, gender, and region). Multicollinearity was checked by evaluating the standard error for each variable in the equation, which showed very small values, implying that the model is statistically stable [28]. Effect sizes with 95% confidence intervals were calculated. The significance threshold was set at *p* ≤ 0.05 for all the statistical tests.

## 3 Results

**FIGURE 1:**
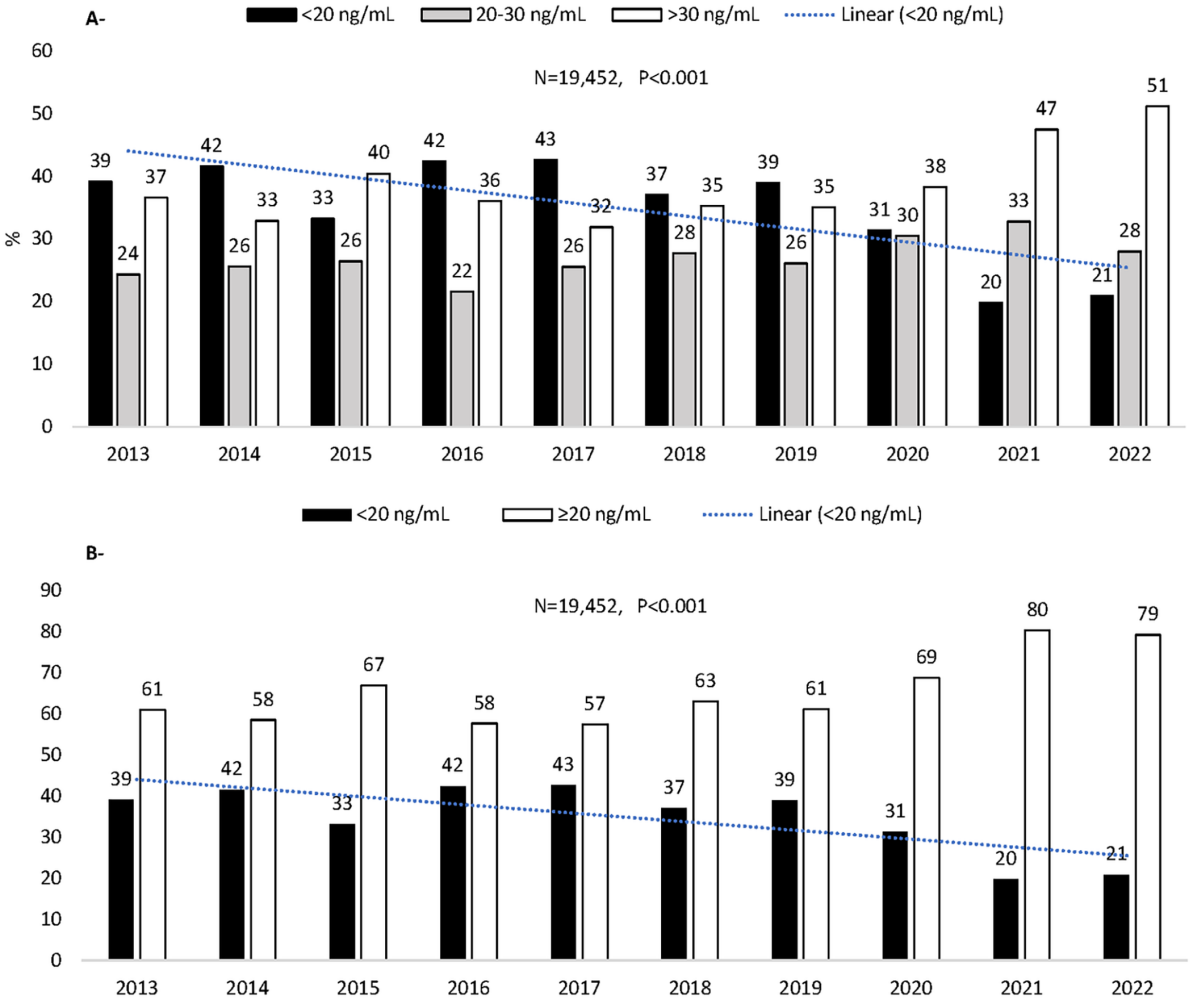
Yearly distribution of Vitamin D levels (%) in the study sample of Lebanese residents. (A) Yearly distribution according to the deficiency (< 20 ng/mL), insufficiency (20–30 ng/mL), and sufficiency levels (> 30 ng/mL). (B) Yearly distribution according to the WHO for the Eastern Mediterranean Region (20 ng/mL).

Our study involved 19,452 Lebanese individuals with different sociodemographic characteristics, all shown in Table 1. The participants' mean age was 53.4 ± 17.6 years. They were divided into four age groups that happened to be of similar proportions (Table 1). Sixty‐five percent of the participants were females. Most participants were from Mount Lebanon (47%) and Beirut (41%). The number of individuals tested for VitD increased significantly, from 1120 in 2013 to 4633 in 2022 (Table 1). The average VitD level was 26 ± 12 ng/mL, with the highest level recorded in 2021 and 2022 (29.3 ± 12.3 and 29.3 ± 12.2 ng/mL, respectively, Table 1). We analyzed the progression of VitD deficiency over the past 10 years, using the two classifications (Figure 1A,B). While VitD deficiency varied between 39% and 43% before 2019, a noticeable decrease was observed after 2019, with VitD deficiency levels as low as 20% and 21% in 2021 and 2022, respectively (Figure 1A,B).

**TABLE 1:** Sociodemographic characteristics of the Lebanese participants by year.

Characteristics	Total (*n* = 19,452)	2013 (*n* = 1120)	2014 (*n* = 946)	2015 (*n* = 1718)	2016 (*n* = 1307)	2017 (*n* = 913)	2018 (*n* = 2072)	2019 (*n* = 2366)	2020 (*n* = 1590)	2021 (*n* = 2787)	2022 (*n* = 4633)
Age (mean ± SD)	53.4 ± 17.6	58.2 ± 17.2	55.3 ± 17.8	50.1 ± 17.6	54.8 ± 18.5	53.9 ± 18.7	54.8 ± 17.6	54.1 ± 18.1	53.3 ± 17.6	53.1 ± 17.3	51.8 ± 16.8
Age categorized, *n* (%)											
18–35	3716 (19)	130 (12)	152 (16)	429 (25)	238 (18)	187 (20)	350 (17)	442 (19)	300 (19)	533 (19)	955 (21)
35–55	6498 (33)	353 (32)	304 (32)	582 (34)	417 (32)	289 (32)	684 (33)	780 (33)	544 (34)	931 (33)	1614 (35)
55–65	3947 (20)	221 (20)	165 (17)	310 (18)	230 (18)	159 (17)	432 (21)	473 (20)	325 (20)	613 (22)	1019 (22)
> 65	5291 (27)	416 (37)	325 (34)	397 (23)	422 (32)	278 (30)	606 (29)	671 (28)	421 (26)	710 (25)	1045 (23)
Gender, *n* (%)											
Male	6848 (35)	278 (25)	282 (30)	542 (32)	393 (30)	347 (38)	702 (34)	925 (39)	637 (40)	1042 (37)	1700 (37)
Female	12,604 (65)	842 (75)	664 (70)	1176 (68)	914 (70)	566 (62)	1370 (66)	1441 (61)	953 (60)	1745 (63)	2933 (63)
VitD levels (ng/mL) (Mean ± SD)	26 ± 12	25.6 ± 12.5	24.2 ± 11.8	23.5 ± 12.9	24.1 ± 13.2	23.1 ± 12.3	24.6 ± 11.1	24.7 ± 12.4	26.0 ± 11.8	29.3 ± 12.3	29.3 ± 12.2
Region, *n* (%)											
Mount Lebanon	9120 (47)	311 (28)	280 (30)	296 (17)	372 (28)	367 (40)	1103 (53)	1279 (54)	820 (52)	1530 (55)	2762 (60)
Beirut	8040 (41)	680 (61)	573 (61)	878 (51)	838 (64)	447 (49)	734 (35)	778 (33)	562 (35)	1000 (36)	1550 (33)
Outside^a^	2291 (12)	129 (12)	93 (10)	544 (32)	97 (7)	99 (11)	235 (11)	309 (13)	208 (13)	257 (9)	321 (7)

*Note:* Values are arithmetic mean ± SD for scale variables. Categorical variables were shown as numbers (*n*) and percentages (%).

Abbreviation: VitD, Vitamin D levels.

^a^ Outside Mount Lebanon and Greater Beirut regions.

We divided our participants according to VitD levels into three groups, as conventionally used for assessing the VitD status (deficient, insufficient, and sufficient), or two groups according to the regional guidelines of the WHO for the Eastern Mediterranean Region (deficient and sufficient) (Tables 2 and 3). In both cases, results showed that 31% were deficient, 28% were insufficient, and 41% were sufficient (Table 2) or 69% were sufficient (Table 3). Overall, when comparing sufficiency in VitD between males and females from all age groups, only 36% of males had VitD levels > 30 ng/mL (*p* < 0.01, Table 2) whereas 65% had ≥ 20 ng/mL (*p* < 0.01, Table 3). This level was significantly higher in females, with 44% (*p* < 0.001, Table 2) versus 71% of sufficiency in VitD (*p* < 0.01, Table 3). Interestingly, a decrease in VitD deficiency was noticed disproportionally with age; thus, the older the participant is, the higher their VitD levels are. While 40% of participants below 35 years old presented with VitD deficiency, this level decreased significantly after 35 years old to 27% and 29%, respectively, in the age group 55–65 and over 65 years old (*p* < 0.001, Tables 2 and 3). Regarding gender differences, females over 65 years old were significantly less prone (25%) than males over 65 years old (34%) to VitD deficiency (Table 2). Lastly, VitD deficiency was significantly lower outside (25%) than in Beirut (35%) and Mount Lebanon (29%) (*p* < 0.01, Tables 2 and 3). Interestingly, females above 55 showed the highest VitD levels, as 51% had VitD levels above 30 ng/mL (Table 2). This proportion increased to 75%–78% when choosing a lower threshold (≤ 20 ng/mL, Table 3). Around 4% of participants had VitD levels below 12 ng/mL. The majority (85%) resided in the Beirut and Mount Lebanon regions, with the lowest values observed during the winter. Only one individual had a VitD level exceeding 200 ng/mL (data available upon demand).

**TABLE 2:** Vitamin D levels in different genders and age groups living in Mount Lebanon, Beirut, and other areas.

Variables	Vitamin D status, n(%)											
	Overall (*n* = 19,452)				Males (*n* = 6848)				Females (*n* = 12,604)			
	Deficient (< 20 ng/mL)	Insufficient (20–30 ng/mL)	Sufficient (> 30 ng/mL)	*p*	Deficient (< 20 ng/mL	Insufficient (20–30 ng/mL)	Sufficient (> 30 ng/mL)	*p*	Deficient (< 20 ng/mL	Insufficient (20–30 ng/mL)	Sufficient (> 30 ng/mL)	*p*
	6049 (31)	5367 (28)	8036 (41)	< 0.01^a^	2405 (35)	1945 (28)	2498 (36)	< 0.01^a^	3644 (29)	3422 (27)	5538 (44)	< 0.01^a^
VitD (mean ± SD)	13.7 ± 4.3	25 ± 2.3	36.9 ± 11.3	< 0.01^a^	13.8 ± 4.1	24.9 ± 2.30	35.7 ± 11.09	< 0.01^a^	13.7 ± 4.4	25. ± 2.3	37.4 ± 11.3	< 0.01^a^
Age (mean ± SD)	50.89 ± 18.4	51.80 ± 17.2	56.43 ± 16.8	< 0.01^a^	53 ± 18.1	52.65 ± 16.9	56.14 ± 17.2	< 0.01^a^	49.50 ± 18.4	51.32 ± 17.3	56.56 ± 16.6	< 0.01^b^
Age categorized, *n* (%)												
18–35	1491 (40)	1117 (30)	1108 (30)	< 0.01^b^	479 (40)	349 (29)	363 (30)	< 0.01^b^	1012 (40)	768 (30)	745 (30)	< 0.01^b^
35–55	1995 (31)	1903 (29)	2600 (40)		771 (33)	709 (31)	831 (36)		1224 (29)	1194 (28)	1769 (42)	
55–65	1045 (27)	1100 (28)	1802 (46)		496 (34)	425 (30)	516 (36)		549 (22)	675 (27)	1286 (51)	
> 65	1518 (29)	1247 (24)	2526 (48)		659 (34)	462 (24)	788 (41)		859 (25)	785 (23)	1738 (51)	
Region, *n* (%)												
Mount Lebanon	2662 (29)	2560 (28)	3898 (43)	< 0.01^b^	1173 (34)	1001 (29)	1284 (37)	< 0.01^b^	1489 (26)	1559 (27)	2614 (46)	< 0.01^b^
Greater Beirut	2811 (35)	2133 (26)	3096 (38)		1012 (39)	693 (27)	899 (34)		1799 (33)	1440 (26)	2197 (40)	
Outside^c^	575 (25)	674 (29)	1042 (45)		220 (28)	251 (32)	315 (40)		355 (23)	423 (28)	727 (48)	

Abbreviation: VitD, Vitamin D levels.

^a^ Kurskal–Wallis test was used to assess the significance between vitamin D levels and age across the three groups.

^b^ *χ* ^2^ test of independence was used to assess the significance between the age categorized, the region versus vitamin D status.

^c^ Outside Mount Lebanon and Greater Beirut regions.

**TABLE 3:** Vitamin D levels in different gender and age groups according to the regional guidelines of the WHO for the Eastern Mediterranean Region (20 ng/mL).

Variables	Vitamin D status, *n* (%)								
	Overall (*n* = 19,452)			Males (*n* = 6848)			Females (*n* = 12,604)		
	< 20 ng/mL	≥ 20 ng/mL	*p*	< 20 ng/mL	≥ 20 ng/mL	*p*	< 20 ng/mL	≥ 20 ng/mL	*p*
	6049 (31)	13,403 (69)	< 0.01^a^	2405 (35)	4443 (65)	< 0.01^a^	3644 (29)	8960 (71)	< 0.01^a^
VitD (mean ± SD)	13.7 ± 4.3	40 ± 10	< 0.01^a^	13.8 ± 4.1	40 ± 9.7	< 0.01^a^	13.7 ± 4.4	40.4 ± 10.2	< 0.01^a^
Age (mean ± SD)	50.89 ± 18.4	57 ± 16.6	< 0.01^a^	53 ± 18.1	57.2 ± 17	< 0.01^a^	49.50 ± 18.4	57.5 ± 16.3	< 0.01^b^
Age categorized, *n* (%)									
18–35	1491 (40)	2225 (60)	< 0.01^b^	479 (40)	712 (60)	< 0.01^b^	1012 (40)	1513 (60)	< 0.01^b^
35–55	1995 (31)	4503 (69)		771 (33)	1540 (67)		1224 (29)	2963 (71)	
55–65	1045 (27)	2902 (73)		496 (34)	941 (66)		549 (22)	1961 (78)	
> 65	1518 (29)	3773 (71)		659 (34)	1250 (66)		859 (25)	2523 (75)	
Region, *n* (%)									
Mount Lebanon	2662 (29)	6458 (71)	< 0.01^b^	1173 (34)	2285 (66)	< 0.01^b^	1489 (26)	4173 (74)	< 0.01^b^
Greater Beirut	2811 (35)	5229 (65)		1012 (39)	1592 (61)		1799 (33)	3637 (67)	
Outside^c^	575 (25)	1716 (75)		220 (28)	566 (72)		355 (23)	1150 (77)	

Abbreviation: VitD, Vitamin D levels.

^a^ Mann–Whitney *U* test was used to assess the significance between vitamin D levels and age across the two groups.

^b^ *χ* ^2^ test of independence was used to assess the significance between the age categorized, the region versus vitamin D status.

^c^ Outside Mount Lebanon and Greater Beirut regions.

We performed a multiple logistic regression to identify the sociodemographic and environmental predictors of VitD deficiency and insufficiency (Table 4). Females had 35% and 28% lower risk of VitD deficiency and insufficiency than males (OR = 0.66, *p* < 0.01; OR = 0.72, *p* < 0.01). Second, living in Beirut significantly increased the risk of developing VitD deficiency or insufficiency by 41% and 15%, respectively (OR = 1.41, *p* < 0.01; OR = 1.15, *p* < 0.01) (Table 4). When compared to the youngest age group (< 35 years), older age groups had a lower risk of developing VitD deficiency and insufficiency, with an OR decreasing respectively from 0.55 and 0.66 for the age group 35–55 years old to 0.41 and 0.53 for age groups above 55 years old (*p* < 0.01, Table 4). Our data also showed that the high and moderate pollution degrees were associated with 33% and 45% higher risk of VitD deficiency than those with low pollution (OR = 1.45, *p* < 0.01; OR = 1.33, *p* < 0.01) (Table 4). Furthermore, the winter and spring seasons were associated with 53% and 64% higher VitD deficiency risk, respectively, when compared with the summer and autumn seasons (OR = 1.53, *p* < 0.01; OR = 1.64, *p* < 0.01) (Table 4).

**TABLE 4:** Multiple regression analysis of gender, age, regional residences, pollution, and seasonal factors in relation to Vitamin D deficiency.

Variables	Vitamin D deficiency	*p*	Vitamin D insufficiency	*p*
	OR (95% CI)		OR (95% CI)	
Age categories				
35–55	0.55 (0.50–0.61)	< 0.01	0.66 (0.6–0.73)	< 0.01
55–65	0.41 (0.37–0.46)	< 0.01	0.53 (0.47–0.59)	< 0.01
Gender				
Female	0.65 (0.61–0.70)	< 0.01	0.72 (0.67–0.78)	< 0.01
Region				
Beirut	1.41 (1.309–1.514)	< 0.01	1.15 (1.069–1.239)	< 0.01
Outside^a^	0.83 (0.742–0.936)	< 0.01	0.91 (0.810–1.018)	0.097
Pollution				
Moderate	1.33 (1.17–1.51)	< 0.01	1.24 (1.1–1.41)	< 0.01
High	1.45 (1.34–1.56)	< 0.01	1.15 (1.07–1.24)	< 0.01
Season				
Autumn	1.01 (0.91–1.13)	0.8	0.99 (0.9–1.1)	0.90
Winter	1.53 (1.4–1.67)	< 0.01	1.04 (0.96–1.14)	0.34
Spring	1.64 (1.49–1.82)	< 0.01	1.04 (0.94–1.16)	0.43

*Note:* The threshold for VitD deficiency was set at 20 ng/mL. The threshold for VitD insufficiency was set at 30 ng/mL.

Abbreviations: CI, confidence interval; OR, odds ratio.

^a^ Outside Mount Lebanon and Greater Beirut regions.

**FIGURE 2:**
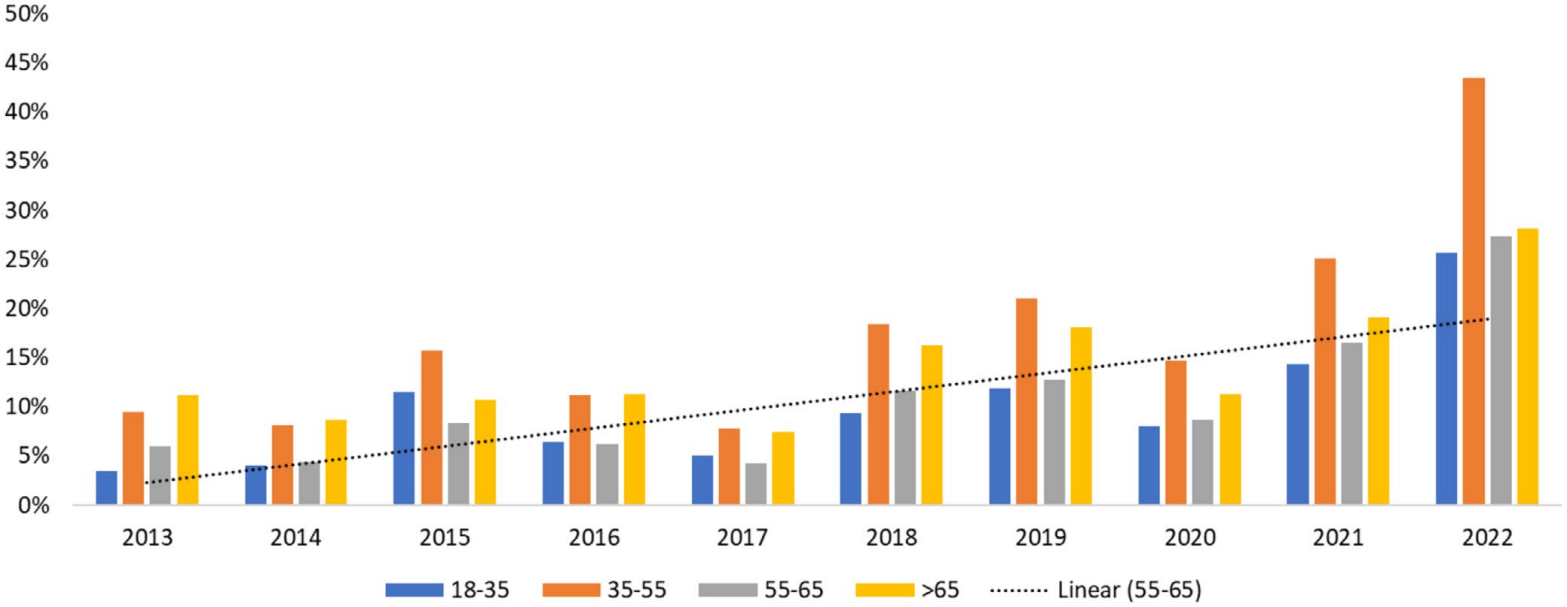
Yearly distribution of individuals tested for Vitamin D levels by age groups. The distribution of the age categories of the individuals testing their Vitamin D levels from 2013 to 2023.

To better shed light on VitD deficiency over the years, we first looked at the yearly distribution of VitD testing according to age groups (Figure 2). No significant variation was observed over the years for the number of tested participants aged 35–55 and 55–65. However, the percentage of tested participants < 35 years old increased over the years (Figure 2), which was correlated to a significant increase in VitD deficiency for this age group from 16% in 2013 to 31% in 2022 (Table 5). The opposite trend was observed in the > 65‐year‐old age group, with a significant decrease in the percentage of VitD deficient participants for this age group from 32% in 2013 to 19% in 2022 (Table 5).

**TABLE 5:** Yearly prevalence of Vitamin D deficiency (< 20 ng/mL) across different age groups in Lebanon.

	Year										*p*
	2013	2014	2015	2016	2017	2018	2019	2020	2021	2022	
Age categorized, *n* (%)											
18–35	69 (16)	79 (20)	116 (20)	123 (22)	103 (26)	185 (24)	231 (25)	130 (26)	159 (29)	296 (31)	< 0.001
35–55	144 (33)	123 (31)	207 (36)	166 (29)	118 (30)	263 (34)	317 (34)	157 (32)	181 (33)	319 (33)	
55–65	82 (19)	56 (14)	97 (17)	86 (15)	58 (15)	131 (17)	170 (18)	101 (20)	93 (17)	171 (17)	
> 65	143 (32)	135 (34)	150 (26)	179 (32)	110 (28)	188 (25)	203 (22)	110 (22)	119 (21)	181 (19)	
Total	438	393	570	554	389	767	921	498	552	967	

*Note:* *p*: A *χ* ^2^ test of independence to test the association between the age groups and the prevalence of vitamin D deficiency.

**FIGURE 3:**
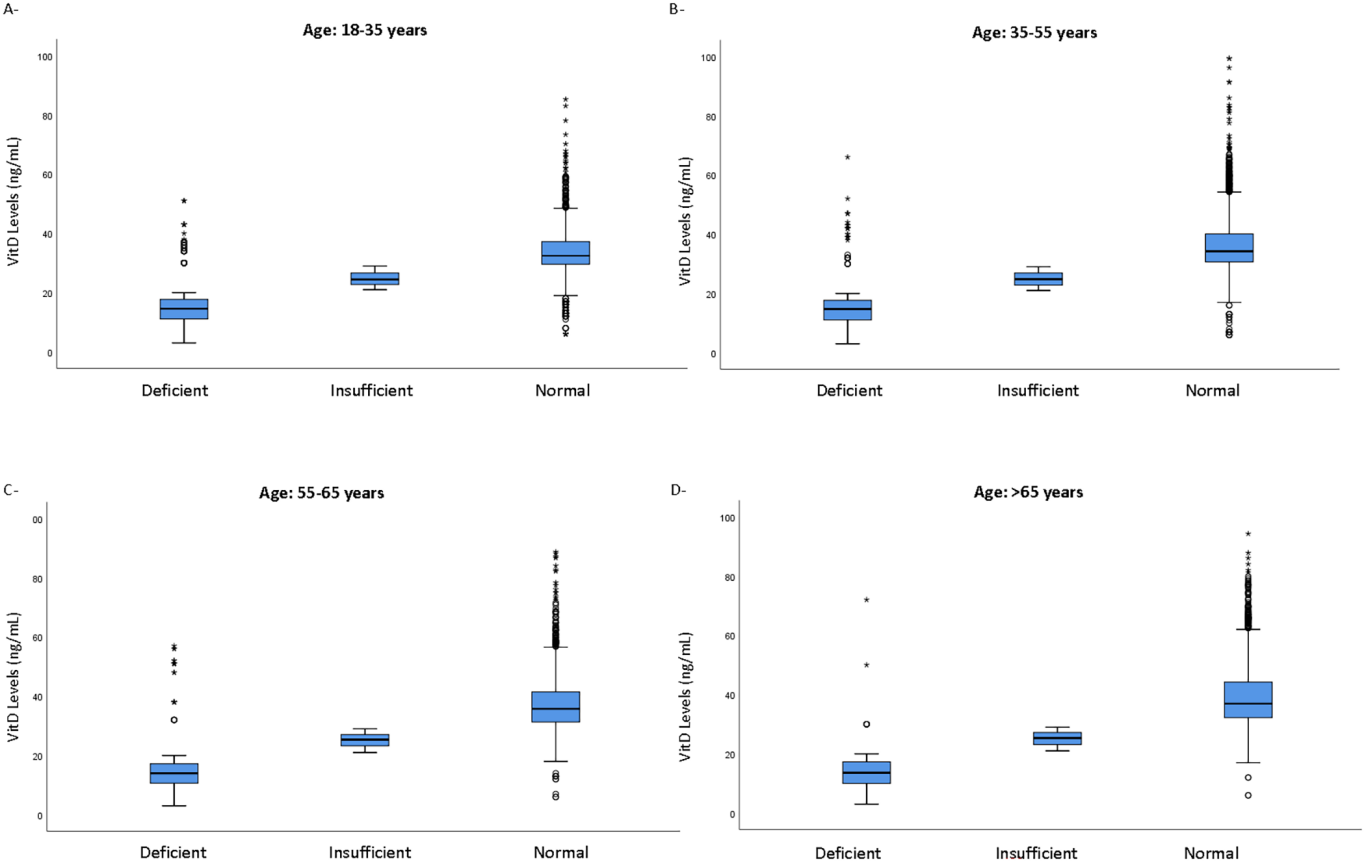
Distribution of Vitamin D levels by deficiency status across different age groups. VitD, Vitamin D.

Using the conventional VitD level classification (three groups: deficient vs. insufficient vs. sufficient), we found across all age groups that individuals with normal VitD status consistently showed higher levels of VitD and were the most represented with significant variability (Figure 3).

## 4 Discussion

Studies show that VitD deficiency is more common in Asia than in Europe, North America, or New Zealand [29]. Specifically, VitD deficiency is highly prevalent in Lebanon [4]. Despite having enough daylight exposure, VitD deficiency is a major public health issue in Lebanon.

Screening for VitD deficiency and prescribing VitD dietary supplementation is especially important in high‐risk populations [8] such as pregnant women [30, 31], post‐menopausal women [32], and persons with clinical evidence of osteoporosis or diseases leading to osteoporosis [33]. In the Middle East and North Africa (MENA) region, several VitD guidelines have been published to encourage physicians to prevent the increase in VitD deficiency effectively [34, 35, 36, 37]. The year 2023 marked the first national initiative by the Lebanese Ministry of Public Health to develop suitable guidelines for administering VitD [38]. Among the key recommendations were promoting healthy eating habits and reasonable sun exposure as sources of VitD.

Our results show that the prevalence of VitD deficiency in the Greater Beirut and Mount Lebanon areas has varied significantly over the past 10 years. These findings raise substantial concerns regarding the quality of public health and healthcare services in Lebanon, as addressing VitD deficiency is relatively straightforward. The high prevalence of suboptimal VitD status underscores a critical public health issue requiring urgent attention and intervention.

In urban areas with high pollution, initiatives to reduce air pollution may indirectly enhance VitD synthesis. Studies have shown that ambient air pollutants can absorb and diffuse solar irradiation, leading to decreased ground levels of UVB, thereby adversely affecting serum VitD levels in the population [39, 40, 41]. This relationship underscores the importance of addressing air quality for maintaining adequate VitD levels. Seasonal considerations could involve encouraging outdoor activities during periods of optimal sunlight and offering subsidized supplements during winter. Outdoor time was directly associated with VitD levels, highlighting that even in regions with high UVB potential, outdoor activity remains a key determinant of VitD status [42]. Tailored interventions, such as outreach through healthcare providers to high‐risk groups like males, younger individuals, and those in polluted areas, can ensure equitable access to resources and promote improved VitD levels across the population.

Using the conventional cutoff values for VitD deficiency, insufficiency, and sufficiency, our data showed that 31% of our participants were deficient, 28% were insufficient, and 41% were sufficient. When using the recent WHO classification for the Mediterranean region, 69% of participants were sufficient and 31% were deficient for VitD overall. Although the prevalence of VitD deficiency was high (31%), it was much lower than the one reported 24 years ago by Gannagé‐Yared et al. (72.8%) [43]. Though our sampling method differs from Gannagé‐Yared et al., the comparison might reflect the successful implementation of awareness campaigns to promote the screening of VitD deficiency in these regions. This is supported by our data showing an increase in the total number of persons tested each year for VitD level. Four factors were associated with the higher risk of VitD deficiency: younger age, male gender, higher pollution level, and city location. The analysis of air quality data revealed that Beirut consistently exhibited the highest pollution levels, followed by Mount Lebanon, with the remaining regions displaying comparatively lower levels. Notably, Beirut is among the most polluted cities globally, underscoring the pressing need for targeted environmental interventions [44].

A significant decrease in VitD deficiency was observed between 2013 and 2022 in the older age groups. A study by Arabi et al. and another by Cui et al. mentioned that VitD deficiency was seen more commonly in females than males [18, 35]. However, our study showed that females over 65 were significantly less prone than males over 65 to VitD deficiency. The higher VitD levels in females could be due to several factors, especially that healthcare providers focus on the importance of measuring VitD routinely in women, as it might lead to osteoporosis. For instance, older females may be more likely to take VitD supplements or adhere to osteoporosis prevention measures due to increased awareness and healthcare interventions targeting postmenopausal women [45, 46]. Additionally, women are typically more proactive in seeking medical attention and monitoring their VitD levels. Factors influencing the prevalence of VitD may vary by gender [47]. This includes hormones such as estrogen, which play a role in the metabolism of VitD [47]. Estrogen may impact how VitD is metabolized and absorbed, leading to higher levels of the nutrient in the blood in women than in men. Conversely, participants younger than 35 were less tested for VitD level and presented with an increased prevalence of VitD deficiency. This aligns with results published by Saad et al. [48], showing that in Lebanon, younger age and male sex are predictors of hypovitaminosis D. However, another study done in Italy showed that older populations are at a higher risk of VitD deficiency [49].

In European and Asian populations, genetic factors significantly determine an individual's VitD status [50, 51]. Twin and family‐based studies have reported that circulating VitD concentrations are 43%–70% heritable, indicating a strong genetic influence [51, 52]. Several genetic variants have been identified that are associated with VitD deficiency. Genome‐wide association studies have uncovered multiple single nucleotide polymorphisms (SNPs) in genes involved in VitD metabolism and signaling that are linked to VitD status [50, 51, 53]. The *GC* gene encodes the VitD binding protein, which transports VitD metabolites in the blood. Variations in *GC* have been associated with 25(OH)D levels in different populations [54]. This includes‐ variant‐ *GC* (rs4588, rs7041, rs2282679). Other variants in genes are *CYP2R1* (rs10741657), *DHCR7* (rs12785878), and *VDR* (TaqI, BsmI, FokI) [51, 54, 55]. The *CYP2R1* and *DHCR7* genes are involved in the synthesis of 25(OH)D, the main circulating form of VitD, and their variants have also been linked to VitD status [50, 51]. Additionally, polymorphisms in the *VitD receptor* (*VDR*) gene, which mediates the biological actions of VitD, have been reported to influence VitD levels and its associated health outcomes [55, 56]. For example, the *VDR* BsmI polymorphism has been associated with VitD deficiency and obesity [55]. Genetic factors can also interact with environmental factors to modulate VitD status. Factors such as age, obesity, skin pigmentation, sun exposure, and dietary intake can all influence VitD levels, and their effects may be modified by an individual's genetic background [57, 58]. Furthermore, genetic variation in VitD‐related genes has been implicated in the risk and severity of various diseases, including autoimmune disorders, cancer, and infectious diseases [50, 59, 60]. For instance, VitD deficiency has been linked to an increased risk of multiple sclerosis, and genetic polymorphisms in *CYP2R1* and *CYP27B1* have been associated with multiple sclerosis severity [60, 61].

However, in the Lebanese population, the genetic background has a minor impact on VitD levels. The effects of genetic variants on VitD metabolism, are modest compared to lifestyle factors such as diet and exposure to sunlight [8]. In a study by Fakhoury et al. [62], no association of VitD levels with six specific polymorphisms in VitD‐related genes was observed in 460 Lebanese individuals. Future studies should examine the association of other SNPs with VitD levels and the health status of a larger Lebanese cohort.

When studying the geographic distribution of VitD‐deficient individuals in our population, we noticed they were mainly located in Beirut and Mount Lebanon regions. This is consistent with a study by Arabi et al. [4], which mentioned that elderly people from the community in the Greater Beirut area showed a prevalence of severe VitD deficiency. Living outside the Greater Beirut and Mount Lebanon areas was associated with a decreased risk of VitD deficiency. This aligns with studies showing rural–urban differences in VitD levels [63, 64, 65]. Living outside urban areas is related to lower levels of air pollution and more UVB sunlight, as well as more opportunities for outdoor activities, which can lead to greater sunlight exposure [65].

Although our results might imply an improvement in managing VitD deficiency in the Greater Beirut and the Mount Lebanon regions, these results should be taken cautiously. When looking at the association of age with VitD deficiency, we found that the percentage of tested participants < 35 years old increased over the years, which correlated to a significant increase in VitD deficiency in this age group. This should prompt specialists and healthcare providers to target the younger population. Since the almost complete eradication of the rickets epidemic among children in the 19th century, less and less attention has been given to promoting VitD sufficiency in young age groups [31], with an alarming resurgence of rickets cases reported in 2000 in the United States [66]. A high prevalence of hypovitaminosis D was observed in a large population of 2062 healthy Korean adolescents (13.4%), with a progressive increase as students advanced from elementary school to senior high school [67]. Obesity and physical inactivity have been previously reported as important factors for VitD deficiency [68, 69]. The alarming prevalence of VitD deficiency in individuals younger than 35 should be seriously considered, and appropriate long‐term public health plans should be implemented to encourage sunlight exposure, physical activity, and a rich diet from birth to school to early adulthood.

There are several limitations to our study. (1) The reported decrease in VitD deficiency must be placed in the context of local regulations and ordering policy for VitD testing with a better characterization of the investigated participants, especially since many factors contributing to this deficiency, such as sun exposure, the cultural practices involving clothing that covers most of the skin, and dietary habits, were not considered. One alternative explanation could be that the changes in deficiencies could also result from a dilution effect due to excessive VitD testing in a larger sample size. (2) As the current study is retrospective, it relies on previously recorded data, which may be incomplete or lack certain variables. This limits control over all confounding factors and hinders establishing causality. (3) Potential biases exist, as the data is drawn from a single medical center in Beirut, which may not represent the entire Lebanese population, raising concerns about selection bias. (4) Another limitation is the absence of critical data on participants' dietary habits, sun exposure, and VitD supplementation; all are essential factors in understanding VitD deficiency.

To ensure proper national recommendations are issued, future studies should focus on elucidating the impact of dietary lifestyle and physical activity on the incidence of VitD deficiency, especially in the younger Lebanese population. This will help to optimize VitD intake through diet or supplementation and prevent the risk of VitD‐associated diseases.

## Author Contributions

Conceptualization, S.E.S. and E.S.S.; methodology, P.C.; software, P.C.; validation, S.E.S. and R.F.; formal analysis, S.E.S.; investigation, P.C. and E.S.‐S.; resources, E.S.‐S.; data curation, S.E.S.; writing – original draft preparation, P.C. and E.S.‐S.; writing – review and editing, S.E.S. and R.F.; visualization, S.E.S.; supervision, R.F.; project administration, P.C. All authors have read and agreed to the published version of the manuscript.

## Ethics Statement

Institutional Review Board Statement: The study was conducted in accordance with the Declaration of Helsinki and approved by the Institutional Review Board of Lebanese Hospital Geitaoui University Medical Center (2023‐IRB‐017, August 23, 2023).

## Conflicts of Interest

The authors declare no conflicts of interest.

## Data Availability

For ethical reasons, the data presented in this study are available upon request from the corresponding author.
